# Chiari 1 Malformation in a Child with Febrile Seizures, Parasomnias, and Sleep Apnea Syndrome

**DOI:** 10.1155/2017/8189790

**Published:** 2017-12-17

**Authors:** Marco Zaffanello, Francesca Darra, Tommaso Lo Barco, Francesco Sala, Emma Gasperi, Giorgio Piacentini

**Affiliations:** ^1^Pediatric Division, Department of Surgical Sciences, Dentistry, Gynecology and Pediatrics, University of Verona, Verona, Italy; ^2^Child Neuropsychiatry, Department of Surgical Sciences, Dentistry, Gynecology and Pediatrics, University of Verona, Verona, Italy; ^3^Neurosurgery Section, Department of Neuroscience, Biomedicine, and Movement Science, University of Verona, Verona, Italy

## Abstract

**Introduction:**

The type I is the most common Chiari malformation in children. In this condition, the lower part of the cerebellum, but not the brain stem, extends into the foramen magnum at the base of the skull leading to disturbances in cerebrospinal fluid circulation and to direct compression of nervous tissue.

**Case report:**

We describe a 4-year-old Caucasian female child with febrile seizures, headache, parasomnias, and a delay of speech. The child underwent a magnetic resonance imaging to investigate these neurological signs, disclosing a Chiari malformation type 1. The polysomnography showed a mild-moderate sleep-disordered breathing, increased number of central sleep apneas, and generalized spike waves at sleep onset.

**Conclusions:**

Seizures have been seldom described in CM1 patients. The main reasons for performing MRI in this case were frequent seizures, a delay of speech, and headache, leading to an unexpected diagnosis of CM1. Polysomnography detected a discrete SDB.

## 1. Introduction

The type I is the most common Chiari malformation (CM1) in children with an estimated incidence of 1 : 1000–5000 [1, 2]. The lower part of the cerebellum, but not the brain stem, extends into the foramen magnum at the base of the skull [3]. The neurological signs (headache, neck pain, visual disturbance, vertigo, and ataxia) may suggest such diagnosis [4], lastly confirmed by magnetic resonance imaging (MRI) [1]. This technique can detect an impaired cerebral-spinal circulation and/or a direct compression of the nervous tissue [5]. Idiopathic epilepsy or craniocerebral malformation is an incidental finding in CM1 patients [6, 7].

We describe a child with seizures and parasomnias whose MRI disclosed CM1. We report the night sleep study showing a sleep-disordered breathing (SDB) and discuss the clinical picture taking into count the literature on this topic.

## 2. Case Report

**Figure 1:**
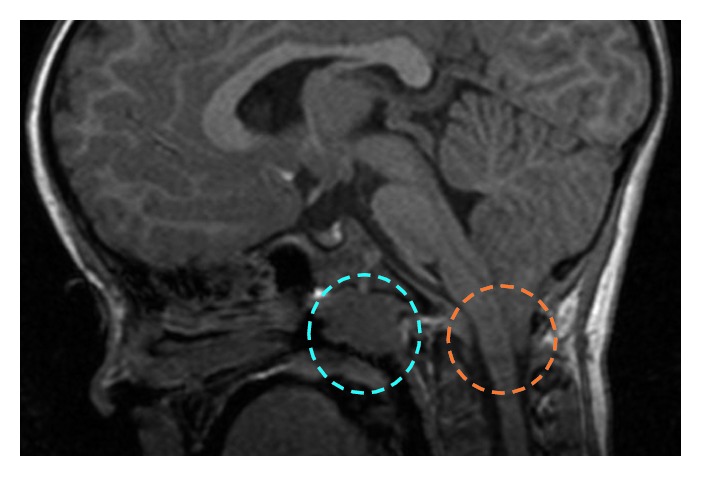
MRI of the brain shows Chiari malformation type 1 with herniation of the cerebellar tonsils beneath the foramen magnum of 17 mm and adenoid hypertrophy.

A Caucasian female child had a family history of febrile seizures in both maternal and paternal line (father's cousin and maternal grandfather's cousin); an older sister had a middle-grade cognitive disability and a disorder of the motor abilities. She was born at-term of uneventful delivery. She reached motor milestones in time; at 2.9 years old, she showed a delay in speech development and febrile seizures. Between the age of 3.3 and 3.8 years, she developed 3 episodes of febrile seizures. From the age of 3.5 years, she presented frequent episodes of a headache localized in the occipital-nuchal region of the skull, sometimes with the nocturnal occurrence that caused reawakening, treated with acetaminophen. She showed frequent night awakenings during which the baby sat on the bed with open eyes and unresponsive to the stimuli or got up and walked around the room with open eyes, producing unintelligible words. The neurological examination showed a delay of speech, but normal cognition (General Development Quotient = 95). The EEG showed good organization of neurological activity and sporadic generalized spikes and wave discharges during drowsiness. To investigate for an occipital headache, at 3.7 years old, she underwent an MRI that disclosed a CM1 with herniation of the cerebellar tonsils beneath the foramen magnum of 17 mm (Figure 1). She was studied with an MRI of the spine which excluded hydrosyringomyelia.

**Figure 2:**
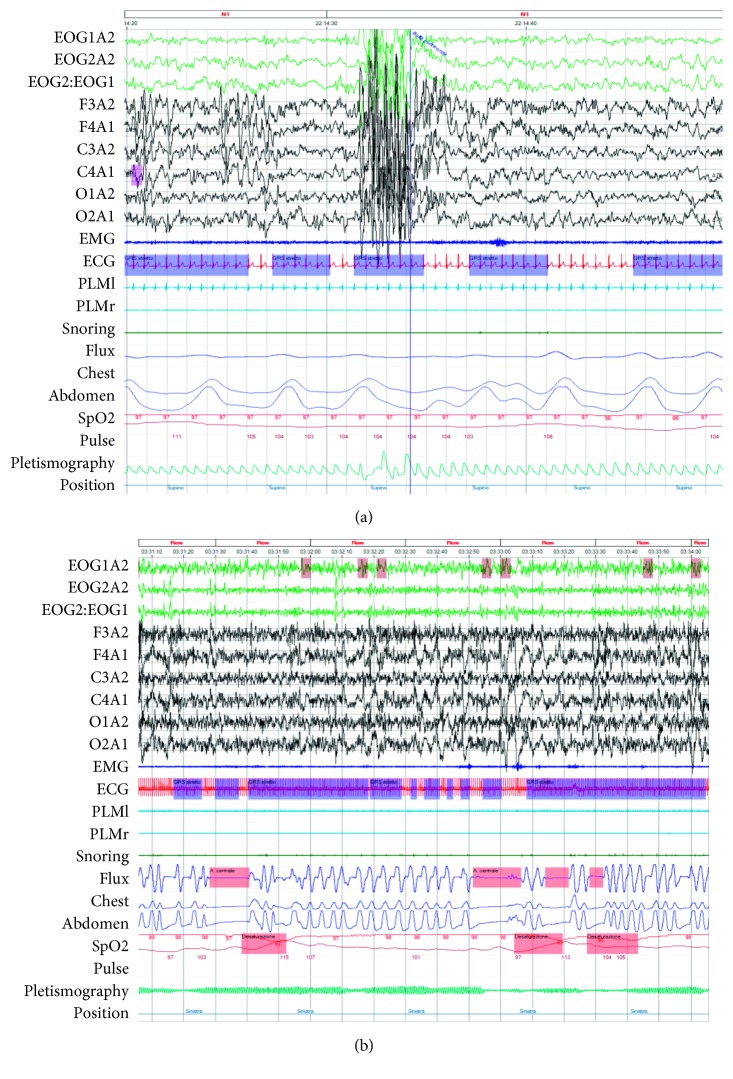
Polysomnography pictures (30 seconds recording) show generalized spike waves during drowsiness (a) and central sleep apneas (3 minutes recording) during the REM stage (b).

At 4 years old (body weight 18 kg (81° percentile), length 107 cm (91° percentile)), the Pediatric Sleep Questionnaire [8] scored positive. During sleep, the parents reported sweating, nightmares, sleepwalking, sleep talking, screams, kicking, and getting out of the bed. Other reported health problems were poor appetite, nasal congestion, difficult nasal breathing, bronchitis, pharyngitis, language problems, seizures, and headaches. From the clinical viewpoint, she showed hypertrophied nasal turbinates, nasal secretions, pale nasal mucosa, slight and moderate obstruction of right and left nostril, tonsillar hypertrophy grade III, Friedman position of palate grade II, open bite, and normal occlusion. To screen for SDB, she underwent a night polysomnography. Sleep respiratory parameters were apnea-hypopnea index (AHI) of 9.9 events/hour, of which obstructive sleep apneas (OSA) of 2.7 events/hour, CSA of 4.7 events/hour, greatest duration of 16 seconds (CSA upper normal value for age of 3.2 events/hour [9, 10]), oxygen desaturation index (ODI) of 3.1 events/hour, basal SatO_2_ of 97%, smallest SatO_2_ of 85%, snoring of 3% of total sleep time (TST), and periodic limb movement (PLM) index of 2/hour. Sleep stages were REM stage of 20.2%, N1 of 7.2%, N2 of 40%, N3–4 of 32.6%, and arousals in REM stage of 5.4 episodes/hour and in the non-REM stage of 6.8 episodes/hour.  Figure 2 shows hypnagogic paroxysmal spike and wave discharges during drowsiness (a) and central sleep apneas (CSA) during the REM stage (b).

The otorhinolaryngology surgeon suggested the adenotonsillectomy and programmed the surgery. We recommended oral melatonin and tryptophan for her disorder of sleep and rectal diazepam at the occurrence of seizures. For the CM1, the neurosurgeon suggested both polysomnography and MRI follow-up.

## 3. Discussion

We described here a 4-year-old Caucasian female child with febrile seizures, headache, parasomnias, and a delay of speech. To investigate for headache recurrences, she underwent an MRI, disclosing CM1. A night polysomnography showed mild-moderate SDB, increased number of CSAs [9, 10], generalized spike waves at sleep onset [11, 12], and poor sleep quality characterized by frequent arousals.

The prevalence of SDB reported in the literature for CM1 was high. Among children and adolescents, the prevalence of SDB by polysomnography varies between 24 and 49% [13, 14]. SDB can be associated with abnormalities in cerebrospinal fluid dynamics in CM1: syringomyelia and hydrocephalus [13]. Obstructive AHI (OAHI) may be associated with tonsillar herniation [14].

AASM reported the role for scoring of CSAs [15]. In addition, Scholle et al. reported the normative values of CSAs in sleep at different age groups and at different stages of maturation [10]. Boudewyns et al. reported that childhood OSA syndrome has CSAs, and adenotonsillectomy leads to a significant decrease of both OSAs and CAI (and resolution of CSAs) [16]. However, patients with medical conditions including neurological disorders and brain stem pathology such as CM1 may have increased CSAs [17]. These disorders involve a disruption or dysfunction of breathing control in the central nervous system [18], likely due to the compression of the structures of the medulla that control the respiration [19]. The relationship between increased central apnea index (CAI) and Chiari malformation is controversial [14, 19]. Children with CM1 and SDB may have an excessive crowding of the brain stem structures at the foramen magnum and a greater length of herniation compared with those children without SDB [1]. Moreover, we described 3 pediatric patients with CM1 [20] who underwent the polysomnography. These patients had OAHI < 1 event/hour and were asymptomatic; only one had had a headache in the clinical history and the increased CSAs [10]. In these cases, the caudal herniation of the cerebellar tonsils, without neurological signs, was not associated with a significantly increased CAI. In summary, children with CSAs and an abnormal neurological examination need neuroimaging [21].

Seizures are seldom described in children with CM1. In particular, Brill et al. reported 11 children with CM1 who presented with seizures, as a marker for subtle cerebral dysgenesis, and a developmental delay in motor or language ability with or without autistic features [22]. So, it is not known if epilepsy and CM1 are related, or if the two disorders are incidentally linked [6]. Elia et al. reported of seven subjects, including only one child, with CM1 and benign seizures of the complex partial type. To explain epileptogenesis, they supposed either cerebral microdysgenesis or a cerebellar dysfunction [23]. So, Iannetti et al. performed an interictal ethylcysteinate-dimer–single-photon emission computed tomography (ECD-SPECT) in 4 children. The hypoperfusion showed by the interictal SPECT scan correlated with EEG focal abnormalities showing cerebral microdysgenesis [24].

In conclusion, we report a CM1 pediatric case in which parasomnias (somnambulism) and recurrent headache are associated with febrile seizures and SDB. A night sleep study by polysomnography can be a suitable method in CM1 patients to assess for both CSAs and OSAs, paroxysmal activity, and sleep quality and to decide for further clinical workup.

## Abbreviations

CAI: Central apnea index
CM1: Chiari 1 malformation
CSAS: Central sleep apnea syndrome
CSAs: Central sleep apneas
OAHI: Obstructive apnea-hypopnea index
ODI: Oxygen desaturation index
OSA: Obstructive sleep apnea
PSQ: Pediatric sleep questionnaire
TST: Total sleep time.
